# Empirical noise mapping guides optimal region‐of‐interest selection for accurate noise estimation in prostate DWI

**DOI:** 10.1002/acm2.70738

**Published:** 2026-09-02

**Authors:** Milica Medved, Aritrick Chatterjee, Ambereen Yousuf, Batuhan Gundogdu, Aytekin Oto, Gregory S. Karczmar

**Affiliations:** ^1^ Department of Radiology The University of Chicago Chicago Illinois USA; ^2^ Sanford J. Grossman Center of Excellence in Prostate Imaging and Image Guided Therapy The University of Chicago Chicago Illinois USA; ^3^ Data Science Institute The University of Chicago Chicago Illinois USA

**Keywords:** diffusion‐weighted imaging (DWI), electronic noise, image reconstruction, magnetic resonance imaging (MRI), prostate cancer

## Abstract

**Background:**

Signal‐to‐noise ratio (SNR) measurements are important for quality assurance, especially when quantitative MRI markers or new hardware are being developed or evaluated. Regions of low signal or assumed uniform signal are used for SNR estimation in a variety of MR image types, but this is unreliable due to spatial heterogeneity of noise arising from the organization of multiple‐detector arrays and sensitivity encoding (SENSE) reconstruction.

**Purpose:**

We present a universally applicable method for empirical noise mapping by propagating simulated noise through the image‐generating reconstruction pipeline, to identify optimal regions of interest (ROIs) for determining noise levels in prostate diffusion‐weighted imaging (DWI).

**Methods:**

Thirty‐six men were imaged at 3T with DWI. K‐space data were propagated through an off‐line reconstruction pipeline including sensitivity encoding (SENSE), to generate DW images. Simulated complex Gaussian noise generated from prescan per‐channel measurements was similarly processed to produce pure noise maps. Per‐voxel standard deviations (SD) of spatial distributions of noise, averaged over prostate, internal obturator muscle (IOM), low signal (anterior and posterior), rectum, and bladder ROIs were calculated and compared to evaluate suitability for SNR estimation in prostate DWI.

**Results:**

IOM ROI noise levels, averaged bilaterally, were not significantly different from those in the prostate. Bladder ROI noise levels were comparable. Noise levels measured in low signal areas were highly dependent on SENSE algorithm regularization strength.

**Discussion:**

The proposed method can be used—on any scanner—to accurately calculate noise levels in MR images, without repeated acquisition or interference from SENSE use.

**Conclusion:**

Bladder and averaged IOM ROIs are appropriate for estimating DWI noise levels in the prostate. Low signal ROIs should not be used, due to the strong dependence on the SENSE regularization strength, and by extension, on the SENSE reconstruction algorithm. The presented method can be used instead to obtain accurate spatial maps of noise levels.

## 1 INTRODUCTION

Prostate cancer is the second most‐common malignancy in men in the United States, with approximately 10% of men being diagnosed with it over their lifetime.^1^ Multiparametric MRI (mpMRI) is a critical step in prostate cancer diagnosis, with diffusion weighted imaging (DWI) and apparent diffusion coefficient (ADC) maps calculated from it being essential diagnostic tools.^2^, ^3^, ^4^ Diagnostic accuracy of DWI and biparametric MRI (T2 and DWI) for prostate cancer varies,^5^, ^6^, ^7^ and improvements are needed for better informed targeted biopsies and ultimate patient outcomes.

In DWI and ADC maps, variability can arise from both electronic noise in the data and physiological and gross anatomic motion. ADC calculations suffer from a downward bias arising from hitting the noise floor at high b values, affecting diagnostic performance when pre‐determined ADC cutoff values are used. Accurate signal noise measurements can help correct for this, but the relevance extends beyond ADC calculations. Efforts to establish quality assurance (QA) processes for prostate mpMRI are underway.^8^, ^9^, ^10^, ^11^ For many specific quantitative quality control (QC) techniques, estimates of electronic noise (primarily from lossy coupling of detector arrays to the body) are needed to accurately estimate added contributions to variability from motion and other artifacts. Further, while the current prostate imaging reporting and data system (PI‐RADS)^12^ relies on the qualitative or visual assessment of DWI and ADC signal intensity, development of standards for quantitative markers such as ADC is an active area, and noise estimates can be essential in that effort.^13^, ^14^, ^15^ Sensitivity‐encoding (SENSE) acceleration is widely used for DWI, both to reduce scan time and to shorten the k‐space readout echo train, which in turn decreases signal loss and spatial distortion. SENSE implementation makes measurements of noise levels in MR images difficult due to a priori unknown spatial heterogeneity of noise inherent to SENSE reconstruction.^16^, ^17^ In prior work, multiple regions with relatively uniform signal have been used for noise estimation, such as the internal obturator muscle (IOM),^18^, ^19^ the bladder,^20^ or regions of low signal in DW images,^19^, ^21^ but this can present problems. IOM signal is not perfectly uniform, and the true heterogeneity of the signal could artificially inflate the perceived noise levels. Bladder can produce uniform signal but is often distant from the structures of interest. Regions of low signal could have artificially and inconsistently suppressed noise levels due to the various regularization methods in the SENSE reconstruction algorithm.^22^, ^23^, ^24^, ^25^, ^26^ Thus, the optimal ROI for signal‐to‐noise ratio (SNR) calculations is not established. However, using the per‐channel electronic noise level information and the spatial profiles of coil element sensitivity—both acquired at the time of the scan and thus available—can yield spatial maps of noise levels, as we demonstrate in this study. These maps can be obtained on a voxel‐by‐voxel level, without repeated measurements or use of calibration phantoms.

The purpose of this study is to present a method—universally applicable on any scanner—for producing accurate spatial noise maps by generating noise‐only k‐space datasets and propagating them through the reconstruction pipeline (including SENSE reconstruction), and to utilize this method to evaluate suitability of several common ROI locations for noise estimation in DWI.

## 2 MATERIALS AND METHODS

### 2.1 Subject population

This study was approved by the University of Chicago Biological Sciences Division Institutional Review Board, in accordance with the Declaration of Helsinki (protocol numbers IRB17‐1694 and IRB18‐1681), and informed consent was obtained from all participants after the nature of the procedures had been fully explained. Thirty‐six men (median age [range]: 64 [48–74] years) scheduled for radical prostatectomy were recruited to receive a pre‐operative mpMRI exam. The participants all had biopsy‐confirmed prostate cancer.

### 2.2 MR imaging

The participants underwent an mpMRI of the prostate on a 3T dStream Ingenia scanner (Philips, Best, Netherlands), with multi‐element anterior and posterior coils and an endo‐rectal coil (ERC) inflated with barium sulfate suspension. The number of coil channels was selected dynamically by the scanner at the time of the scan, with the median number of coil channels employed being 25 (range 20–29). The imaging protocol included standard‐of‐care sequences, including a 2D T2‐weighted (T2W) and a DWI sequence, as well as a DWI‐based hybrid multi‐dimensional MRI (HM‐MRI) series^27^, ^28^, ^29^ that acquired MR images over a 4 × 4 matrix of TE and b values. The data from the HM‐MRI sequence was used for this study, but the principles are applicable to any MRI sequence. HM‐MRI was implemented as a single‐shot spin‐echo echo‐planar imaging (SE‐EPI) sequence with diffusion weighting (field of view 180 × 180 × 102 mm^3^, resolution 1.4 × 1.4 × 3 mm^3^, number of slices 24–34, slice gap 0 mm, SENSE factor 2.2, partial Fourier factor 0.6, fold‐over direction anterior/posterior, b‐values(number of averages per direction) (0(2)/150(1)/1000(1)/1500(4) s/mm^2^; TR 5000 ms; TE 57/70/150/200 ms, SPAIR fat suppression). The TE = 200 ms DWI sequence data was used for this study for the best comparison between the simulated noise maps and in vivo images.

### 2.3 MR image reconstruction and noise map generation

**FIGURE 1:**
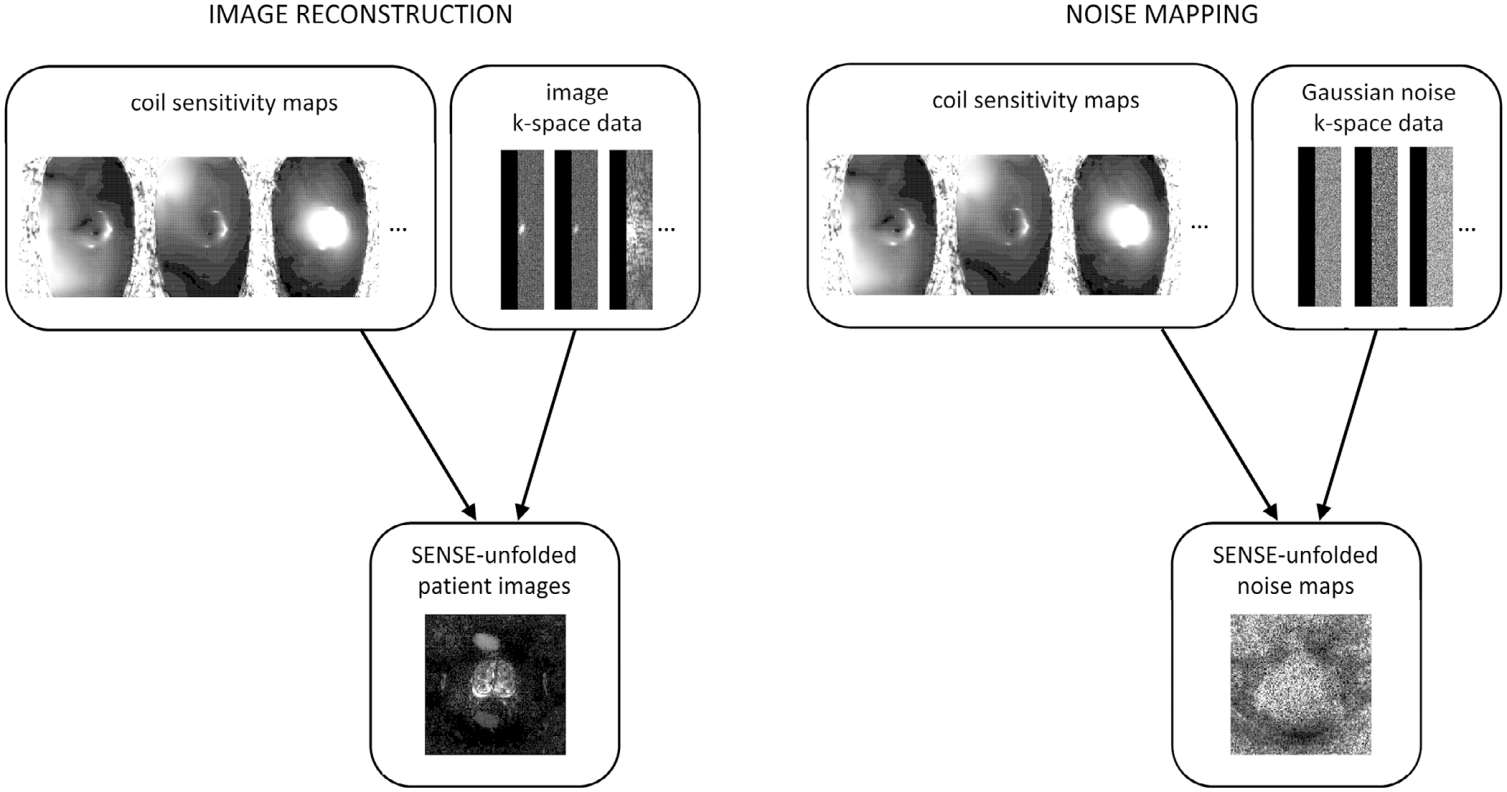
The process of generating diffusion weighted images and noise maps. (LEFT) The complex coil sensitivity maps and partial Fourier complex k‐space data acquired for each coil channel are used to produce SENSE‐unfolded MR images. (RIGHT) When the complex k‐space data is replaced with pure complex Gaussian noise and the same image reconstruction pipeline is used, noise maps are obtained. The standard deviation of the introduced Gaussian noise varies for each coil channel and is determined from actual per‐channel pure noise data collected at the time of imaging.

To facilitate off‐line processing, the raw per‐channel HM‐MRI data, data from reference scans and coil sensitivity scans, and per‐channel noise measurements were captured using the ReconFrame patch (Gyrotools, Zurich, Switzerland). The data were post‐processed off‐line using the MRecon Matlab library (v5.0.13; Gyrotools) that parallels the Philips on‐scanner MR image reconstruction pipeline. Specifically, for DWI‐based HM‐MRI, raw per‐channel signal was first reconstructed into phase‐corrected k‐space data. Second, this partial‐Fourier k‐ space data was further processed to obtain MR images using the SENSE reconstruction algorithm and coil sensitivity data. This is illustrated in Figure 1, left. DW images obtained at the highest TE and b (TE = 200 ms, *b* = 1500 s/mm^2^) values were averaged over 3 directions and 4 averages to produce the final trace image, but all 12 individual directional DW images were also recorded. The DW images were reconstructed to a 128 × 128 matrix (1.4 × 1.4 mm^2^ in‐plane resolution).

A similar process was used to obtain noise maps. The scanner‐provided sequence‐specific, per‐channel noise measurements, in the form of a series of 19920 individual measurements in each readout channel, acquired without RF excitation or gradient engagement. We confirmed that they followed a complex Gaussian distribution, and calculated channel‐specific standard deviations (SDs). These noise measurements provided at the time of the scan were processed to determine the coil element‐specific noise SDs and these were used to generate simulated k‐space datasets for each coil element channel, containing only complex Gaussian noise equivalent to the noise present during image acquisition. These ‘pure noise’ k‐space datasets were processed through the second part of the MRecon pipeline, which produced ‘pure noise’ images, as illustrated in Figure 1, right. To allow for statistical analysis, the noise maps were generated using 16 iterations, with each iteration populating 12 independent individual k‐space instances (three directions and four averages at high b), for a total of 192 noise map samples per participant, per slice, per voxel. Thus, each voxel produced 192 independent noise datapoints, allowing mapping of the SDs of the empirically obtained noise distributions, as expected in each DW image.

Directly measuring the in vivo noise SD maps would require prohibitively long scans. Instead, we considered the 3 × 3 neighborhood of each voxel as exhibiting approximately constant noise levels. Using the highest b, highest TE signal in the 3 × 3 neighborhood, across 3 directions and 4 averages, gave us 108 samples per voxel for estimating noise SD. Using the mid‐gland slice as representative, we correlated the simulated and in vivo SD maps in the areas lateral to the prostate, providing a range of noise levels and avoiding residual signal in the prostate.

To examine the effect of SENSE regularization, the ‘pure noise’ map generation was repeated for four different values of the SENSE reconstruction regularization factor (S‐RF): 0.40, 0.80, 1.20, and 1.41 (default), which illustrated the effect of S‐RF on noise levels in reconstructed images. Unless otherwise indicated, S‐RF = 1.41 was used.

### 2.4 Data analysis

**FIGURE 2:**
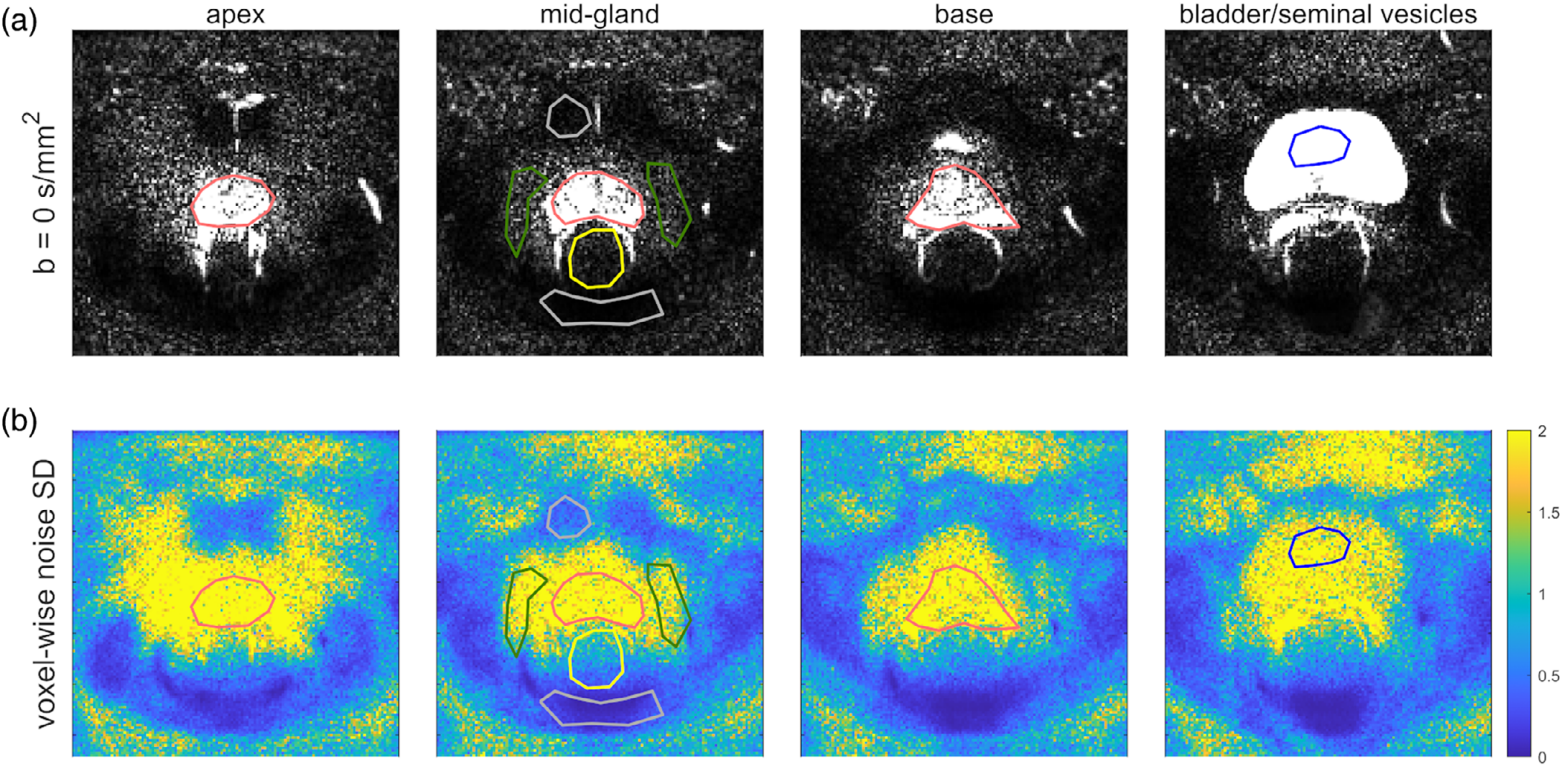
Region of interest (ROI) placement and use to calculate noise SD values. (Top) *b* = 0 s/mm2, TE = 200 ms diffusion weighted images, scaled to best demonstrate the extent of the prostate, are shown at four different levels in the prostate and bladder: apex, mid‐gland, base, and bladder and seminal vesicles. ROIs are outlined in the prostate at three different levels (apex, mid‐gland, base; red), in two areas of low signal (anterior and posterior; gray), in the internal obturator muscle (left and right; green), in the rectum (yellow) and in the bladder (blue). (Bottom) Maps of standard deviation (SD, arbitrary scale) of signal distribution obtained from 192 iterations with simulated noise are shown in the same slices as in the top row, with corresponding ROIs outlined. The SD maps are not symmetrical, and areas of low signal correspond to areas where noise is suppressed by the reconstruction algorithm.

Nine regions of interest (ROIs) were defined on *b* = 0 s/mm^2^ DW images: the apex, mid‐gland, and base of prostate, two areas of low signal (LS, anterior and posterior to the prostate), the left and right IOM, the endorectal coil cross‐section in the rectum, and in the center of the bladder, as illustrated in Figure 2. The averages of the noise SD maps, ⟨NSD⟩, over the defined ROIs were calculated. In addition, the average SD over the three prostate ROIs was calculated. To demonstrate the asymmetry of the noise maps, the IOM ROIs were grouped into “lower signal” and “higher signal”, rather than “left” and “right”. This demonstrated the necessity of using the average SD over both IOM ROIs, which was then calculated. Thus 6 noise level measurements were compared: ${\left\langle NSD\right\rangle }_{prostate}$, ${\left\langle NSD\right\rangle }_{IOM}$, ${\left\langle NSD\right\rangle }_{LSant}$, ${\left\langle NSD\right\rangle }_{LSpost}$, ${\left\langle NSD\right\rangle }_{rectum}$, and ${\left\langle NSD\right\rangle }_{bladder}$. In addition, to account for differences in scanner gain settings between exams, ${\left\langle NSD\right\rangle }_{ROI}$ values were also normalized to ${\left\langle NSD\right\rangle }_{prostate}$.

### 2.5 Statistical analysis

In vivo and simulated noise SD maps were compared using the Pearson correlation coefficient for each case individually, and the mean and standard deviation of the correlation coefficients across all cases was reported. Means and inter‐quartile ranges (IQRs) over the subject population were calculated for ${\left\langle NSD\right\rangle }_{ROI}$ values. Lin's Concordance Correlation Coefficient (CCC) was calculated to assess agreement between ${\left\langle NSD\right\rangle }_{prostate}$ and ${\left\langle NSD\right\rangle }_{ROI}$ in other ROIs. CCC was tested for superiority to 0.50 using a one‐sided z‐test, with bootstrapping (*N* = 1000). Normalized ${\left\langle NSD\right\rangle }_{ROI}$ were compared using the Wilcoxon signed‐rank test. All levels for statistical significance were set to 0.05.

## 3 RESULTS

Voxel‐wise noise SD maps shown in Figure 2 are highly non‐uniform, as expected, and generally not symmetrical. They show localized regions of high noise amplification, typically laterally to the endorectal coil and on one side predominantly. This was true even when the endorectal coil was placed with proper alignment to the prostate (with bilateral symmetry).

**FIGURE 3:**
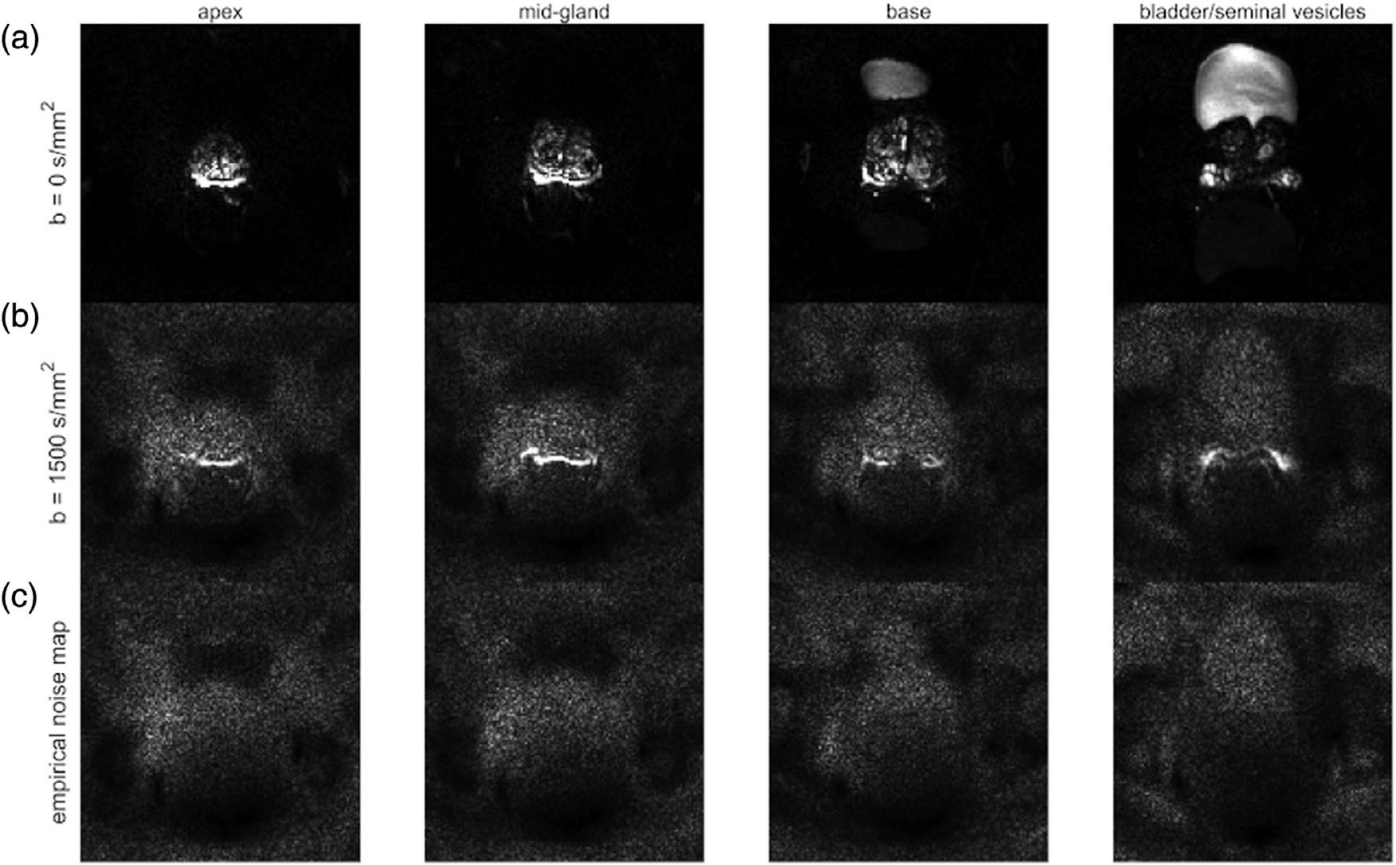
Comparison of high b‐value and “pure noise” images. For a representative exam, four slices at varying levels of the prostate (apex, mid‐gland, base, and bladder/seminal vesicles), (a) *b* = 0 s/mm2 diffusion weighted (DW) images, (b) *b* = 1500 s/mm2 DW images, and (c) corresponding‐slice empirical pure noise maps as reconstructed for the same number of averages as the *b* = 1500 s/mm2 images, are shown. (b) and (c) are shown with the same scaling. The spatial pattern of noise suppression and amplification is similar between (b) and (c). TE = 200 ms in these images.

Figure 3 compares the noise levels and distribution between reconstructed low‐signal DW images and simulated noise‐only DW images. It shows, for a representative MR exam, actual reconstructed DW images at four levels of the prostate, seminal vesicles, and bladder, for TE = 200 ms and *b* = 0 s/mm^2^ (a, for reference), *b* = 1500 s/mm^2^ (b, for comparison with ‘pure noise’ reconstruction) and representative empirically obtained noise maps, obtained with the same number of individual reconstructions (3 directions x 4 averages) as the *b* = 1500 s/mm^2^ DW images (c). Excellent correspondence in spatial distribution of noise is observed between (b) and (c), indicating that the spatial heterogeneity in noise‐only areas in high TE/high b‐value images arises predominantly through electronic noise propagation and validating our approach. The Pearson correlation coefficient between simulated and in vivo noise SD maps was 0.82 ± 0.05, indicating good agreement.

**FIGURE 4:**
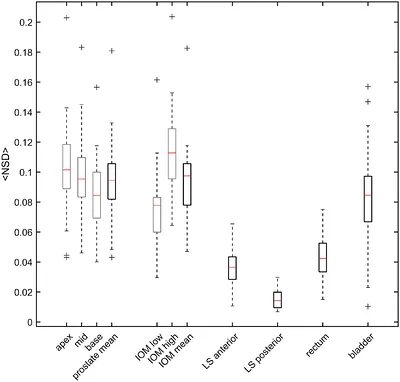
Boxplots of ${\left\langle NSD\right\rangle }_{ROI}$, as obtained from simulations. The values in the prostate apex, mid‐gland, and base are shown in gray, as they are not used individually in data analysis. The values in internal obturator muscle (IOM) for the sides with lower and higher intensity, are similarly shown in gray, as they are not used individually. The areas of low signal (LS) anterior and posterior to the prostate, the rectum, and the bladder are also shown.

**FIGURE 5:**
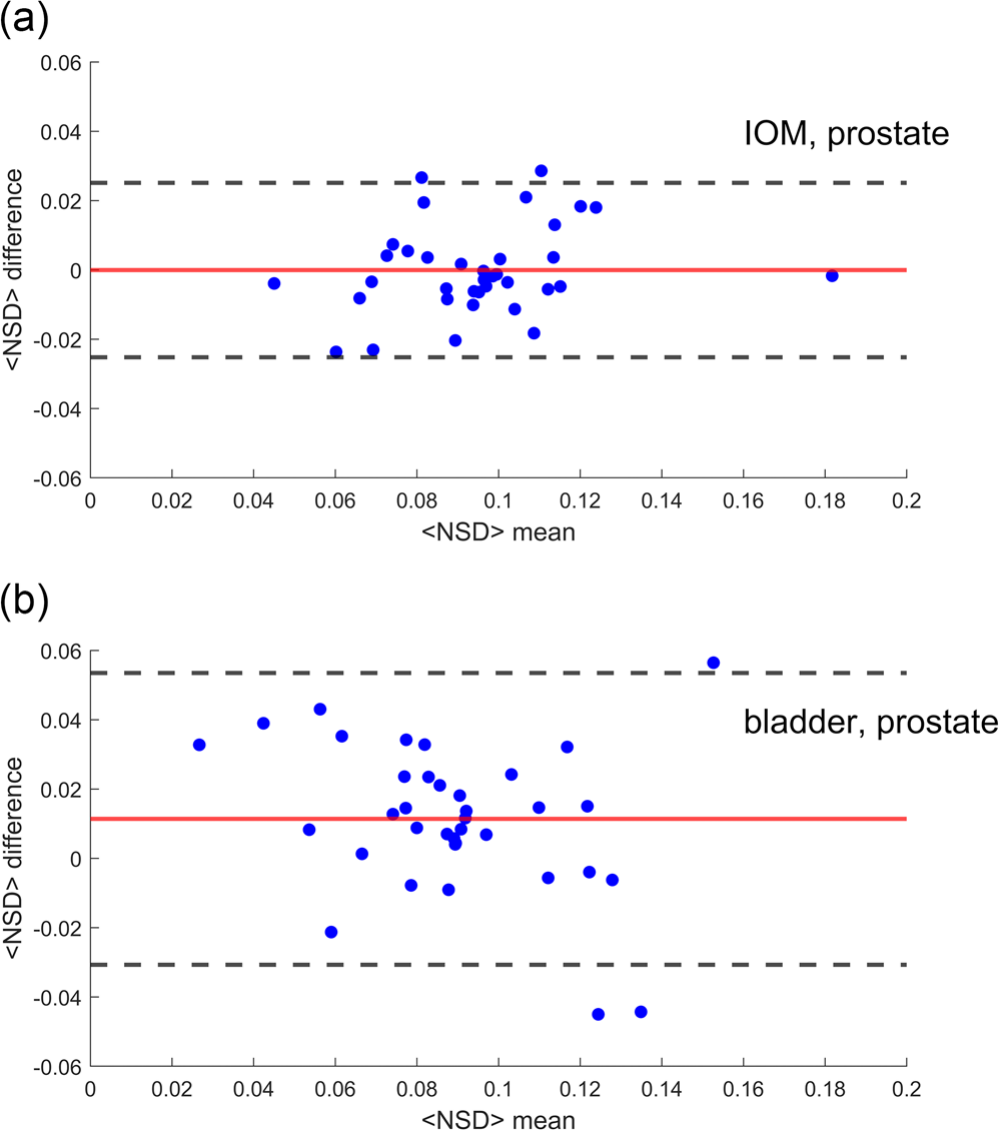
Bland‐Altman analysis of (a) ${\left\langle NSD\right\rangle }_{IOM}$) and (b) ${\left\langle NSD\right\rangle }_{bladder}$, in comparison to ${\left\langle NSD\right\rangle }_{prostate}$. Both regions of interest show good agreement with prostate values.

Figure 4 shows the boxplots of average ${\left\langle NSD\right\rangle }_{ROI}$ for all defined ROIs, for comparison to the mean over the three prostate ROIs. There is high consistency between the three prostate ROIs, and the ${\left\langle NSD\right\rangle }_{prostate}$ is well approximated by the ${\left\langle NSD\right\rangle }_{IOM}$. ${\left\langle NSD\right\rangle }_{bladder}$ shows similar, though slightly lower values. The signal was higher in the left IOM in 83% of the cases, and in the right IOM in 17% of the cases. Table 1 shows the Lin's CCC values for various ${\left\langle NSD\right\rangle }_{ROI}$. The Lin's CCC for ${\left\langle NSD\right\rangle }_{IOM}$ and ${\left\langle NSD\right\rangle }_{bladder}$ were statistically significantly greater than 0.50 (*p* < 0.01). Figure 5 shows Bland‐Altman analysis of ${\left\langle NSD\right\rangle }_{IOM}$ and ${\left\langle NSD\right\rangle }_{bladder}$, in comparison to ${\left\langle NSD\right\rangle }_{prostate}$, with both IOM and bladder ROIs showing good agreement with prostate values.

**TABLE 1:** Lin's CCC between ${\left\langle NSD\right\rangle }_{ROI}$ values and ${\left\langle NSD\right\rangle }_{prostate}$.

ROI	Lin's CCC
IOM	0.86^*^
low signal ROI (anterior)	0.06
low signal ROI (posterior)	0.00
rectum	0.12
bladder	0.67*

Abbreviations: IOM, internal obturator muscle; Lin's CCC, Lin's Concordance Correlation Coefficient; ROI, region of interest;

* statistically significantly greater than 0.50.

**FIGURE 6:**
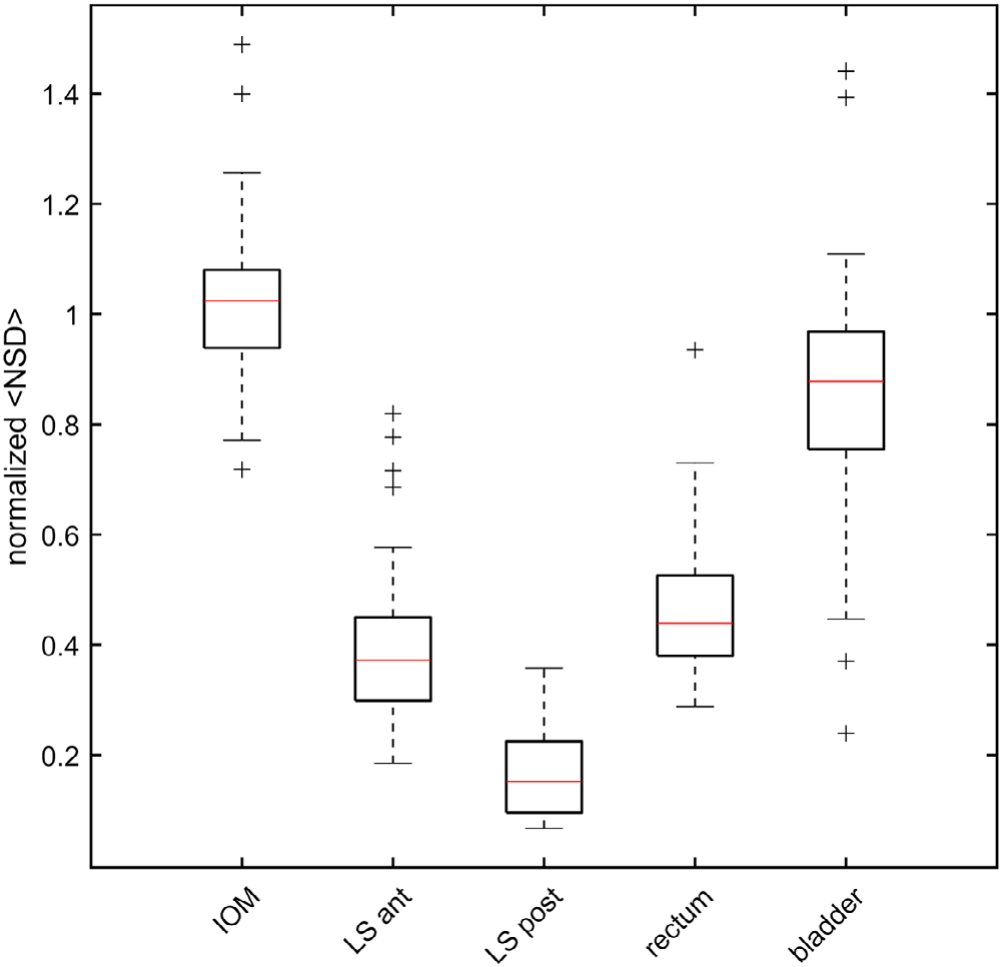
Boxplots of normalized ⟨NSD⟩. Boxplots of normalized ⟨NSD⟩ are shown for interior obturator muscle (IOM), anterior and posterior low signal (LS) regions of interest, rectum, and bladder.

To account for differences in scanner gain between scans, in Figure 6, we are showing the boxplots of normalized ${\left\langle NSD\right\rangle }_{ROI}$ for IOM, low signal regions, rectum, and bladder. This approach facilitates visualization of the inter‐subject variability. The IOM ROI shows excellent agreement with the prostate ROI, while the bladder ROI shows similar, though slightly lower noise levels. Table 2 shows the normalized ${\left\langle NSD\right\rangle }_{ROI}$ for the ROIs shown in Figure 6 and the corresponding IQRs, for different S‐RF settings. ${\left\langle NSD\right\rangle }_{ROI}$ values in all ROIs except IOM were statistically different from ${\left\langle NSD\right\rangle }_{prostate}$.

**TABLE 2:** Normalized ${\left\langle NSD\right\rangle }_{ROI}$ means and corresponding IQRs, as a function of S‐RF.

S‐RF	1.41^a^	1.20	0.80	0.40
Normalized averaged SD means, per ROI				
IOM	1.02^*^	1.08	1.19	1.29
low signal ROI (anterior)	0.40	0.49	0.76	1.21
low signal ROI (posterior)	0.17	0.20	0.27	0.36
Rectum	0.46	0.46	0.45	0.45
Bladder	0.87	0.93	1.05	1.16
Normalized averaged SD IQRs, per ROI				
IOM	0.14	0.15	0.18	0.24
low signal ROI (anterior)	0.15	0.17	0.24	0.39
low signal ROI (posterior)	0.13	0.15	0.20	0.28
Rectum	0.15	0.15	0.15	0.16
Bladder	0.21	0.24	0.40	0.48

Abbreviations: IOM, internal obturator muscle; IQR, interquartal range; ROI, region of interest; SD, standard deviation; S‐RF, SENSE reconstruction regularization factor;

^a^ default value for S‐RF.

* not statistically significantly different from 1 (only S‐RF = 1.41 was tested).

## 4 DISCUSSION

Regions of low signal or regions assumed to have uniform signal are sometimes used for noise estimation in DWI and derived ADC maps, but this method is unreliable. This is due to the spatial heterogeneity of noise levels resulting from the variable spatial sensitivity of coil elements, the noise amplification due to the geometric factor g inherent in reconstruction of SENSE‐accelerated acquisitions, and the noise suppression due to various regularization schemes used in SENSE reconstruction. An empirical method for calculating the spatial distribution of noise would allow reliable measurements of the signal to noise ratio for each region or anatomical feature in the prostate. Here, we present such a method, that can be used to map the electronic noise in the acquired images on a voxel‐by‐voxel basis, without repeated measurements or use of calibration phantoms, and that can be practically and easily implemented on any scanner. Both the necessary excitation‐free per‐channel measurements and the reconstruction pipelines are already implemented on modern commercial scanners.

The method presented here uses the channel‐wise measurements of electronic noise and coil sensitivity that are routinely acquired during the MR exam. This information is used to simulate ‘pure noise’ k‐space data that is then propagated through the usual reconstruction pipeline, to produce ‘pure noise’ MR images. We show that the spatial noise maps obtained in this way correspond to the noise levels observed in actual MR images, validating our approach. We are not the first to propose propagation of simulated noisy k‐space datasets.^30^, ^31^ However, rather than characterizing the g‐factor of the coil configuration, our implementation is applied to in vivo prostate DWI datasets to evaluate suitability of various commonly used ROIs for estimation of prostate noise levels when raw data is not available. Importantly, the per‐channel noise measurements would allow calculation of noise maps even without acquisition of high‐TE, high b‐value DWI. Here, this data was used only for validation of simulated noise maps. The presented method would allow direct calculation of noise levels in the ROI of interest (e.g., prostate or a lesion), without need for defining reference ROIs. In absence of its implementation, we can use appropriate reference ROIs, as identified here.

In this application to prostate MRI exams using an endorectal coil, we find that noise levels are relatively consistent at different levels of the prostate (apex, mid‐gland, base), and that the average of the two IOM ROIs provides the best match to the prostate noise levels, as evidenced by the high CCC. It must be noted that, due to the strong asymmetry in the noise spatial profiles, an average of the two IOM ROIs (left and right) must be used. The bladder ROI also provides a close estimate of prostate DWI noise levels when an endorectal coil is used. While the fidelity of the bladder ROI‐derived noise level estimates is somewhat lower, this is balanced by the advantage of bladder signal decreasing quickly at high b values, and the calculated SD in the bladder ROI being dominated by noise. Conversely, in IOM ROIs, residual signal could be present and the non‐uniformity of the residual tissue signal could artificially inflate the calculated SD in the ROI. While both ROIs show increased normalized SD with lower S‐RF values, the IOM is closer to prostate values at the default S‐RF = 1.41 value, and has lower variability, as evidenced by the lower IRQ. Thus, depending on application, IOM might be more or less appropriate than the bladder as the reference ROI.

Further, the rectum is often used to estimate noise levels, especially when an endorectal coil is used, but this should be avoided. While the normalized mean SDs in the rectum have a low IQR (good consistency), they are underestimating the noise levels in the prostate by approximately a factor of two. While consistency can be of high value in some applications, it is not clear that the correction factor would remain stable for different reconstruction methods. Similarly, the low signal regions highly underestimate the noise levels found in the prostate and, in addition, can be inconsistent depending on location or the choice of the regularization factor, and by extension, on the choice of the regularization method.

The noise level information is crucial for quality control methods that filter data based on measured signal,^16^, ^32^ or for estimation of confidence intervals on quantitative MR markers such as ADC or those derived from intravoxel incoherent motion (IVIM) modeling, which are sensitive to noise.^33^, ^34^, ^35^, ^36^ Measurements of these parameters are biased when the noise floor is not taken into account, and the resulting error can be significant and may affect cancer detection. The noise level information is also important in any application that evaluates image quality in the prostate or elsewhere, such as when a novel coil or reconstruction method are being evaluated for performance.^37^, ^38^, ^39^, ^40^, ^41^ Additional image processing methods such as those relying on principal component analysis (PCA)^36^, ^42^, ^43^, ^44^, ^45^ or singular value decomposition (SVD) more generally, could also benefit.

While the focus of this paper was on prostate DWI using an ERC, the method presented here is of universal utility. It can be implemented on any scanner to provide spatial maps of noise that can be delivered simultaneously with anatomical images, for application to scanner performance testing or quantitative MR research. This can be done, in principle, for any MR image type, without repeated measurements or phantom placement. Other applications may include pulse sequence development, where a measurement of the electronic noise levels may help identify additional image corruption due to stimulated echoes.

### 4.1 Limitations

There are some limitations to this study. While the method is generally widely applicable, the ROI evaluation results are specific to prostate DWI obtained with an ERC. While technical research is often done using an ERC, the current movement in clinical practice is away from use of ERC and towards using surface array coils only. Thus, an evaluation of appropriate ROIs for noise estimation should be done for exams done without an ERC, using the same methodology. However, it is already clear that the low signal regions will not be an adequate choice. Further, other MR sequences such as T2‐weighted fast spin echo should also be evaluated for potential bias, as the reconstruction pipeline may differ slightly. Finally, our method utilizes a suite of Matlab libraries designed for off‐line reconstruction of raw data, and implementing this method directly on the scanner console would be preferable as it would allow distribution of noise level maps simultaneously with anatomical images, facilitating QA processes and clinical research.

## 5 CONCLUSION

In conclusion, we demonstrated a method for calculating the spatial distribution of MR signal noise levels using data that are already routinely acquired by the scanner at the time of the exam, without repeated measurements or use of calibrating phantoms, and applied it to a set of in vivo prostate DWI exams. We evaluate suitability of various frequently used ROIs for estimation of DW image noise levels and find that the internal obturator muscle, when averaged bilaterally, and the bladder can serve adequately, while use of regions of low signal would highly underestimate noise levels. With modern hardware, the presented method can be implemented directly on the consoles of MRI scanners and can be used in technical development of quality control in DWI, or for image quality assessment when novel hardware or image reconstruction methods are evaluated.

## AUTHOR CONTRIBUTIONS

**Milica Medved**: Conceptualization; methodology; software; validation; formal analysis; investigation; data curation; writing—original draft preparation; writing—review and editing; visualization. **Aritrick Chatterjee**: Investigation; data curation; writing—review and editing. **Ambereen Yousuf**: Investigation; writing—review and editing. **Batuhan Gundogdu**: Investigation; writing—review and editing. **Aytekin Oto**: Resources; writing—review and editing; supervision; project administration; funding acquisition. **Gregory S. Karczmar**: Resources; writing—review and editing; supervision; project administration; funding acquisition.

## CONFLICT OF INTEREST STATEMENT

Milica Medved is the Principal Investigator on a project partially funded by GE Healthcare. Aritrick Chatterjee, Aytekin Oto, and Gregory Karczmar hold equity in QMIS LLC. Milica Medved, Aritrick Chatterjee, Batuhan Gundogdu, Aytekin Oto, and Gregory Karczmar have patent applications on a specialized prostate MR imaging technique. Aytekin Oto is on the medical advisory boards of Profound and Akumin.

## ETHICS STATEMENT

This study was approved by the University of Chicago Biological Sciences Division Institutional Review Board, in accordance with the Declaration of Helsinki (protocol numbers IRB17‐1694 and IRB18‐1681), and informed consent was obtained from all participants after the nature of the procedures had been fully explained.

## Data Availability

The data that support the findings of this study are available on request from the corresponding author. The data are not publicly available due to privacy or ethical restrictions.
